# Topologically tuned terahertz confinement in a nonlinear photonic chip

**DOI:** 10.1038/s41377-022-00823-7

**Published:** 2022-05-23

**Authors:** Jiayi Wang, Shiqi Xia, Ride Wang, Ruobin Ma, Yao Lu, Xinzheng Zhang, Daohong Song, Qiang Wu, Roberto Morandotti, Jingjun Xu, Zhigang Chen

**Affiliations:** 1 The MOE Key Laboratory of Weak-Light Nonlinear Photonics, TEDA Institute of Applied Physics and School of Physics, Nankai University, Tianjin, 300457 China; 2 Innovation Laboratory of Terahertz Biophysics, National Innovation Institute of Defense Technology, 100071 Beijing, China; 3 Collaborative Innovation Center of Extreme Optics, Shanxi University, 030006 Taiyuan, Shanxi China; 4 INRS-EMT, 1650 Blvd. Lionel-Boulet, Varennes, QC J3X 1S2 Canada

**Keywords:** Micro-optics, Terahertz optics

## Abstract

Compact terahertz (THz) functional devices are greatly sought after for high-speed wireless communication, biochemical sensing, and non-destructive inspection. However, controlled THz generation, along with transport and detection, has remained a challenge especially for chip-scale devices due to low-coupling efficiency and unavoidable absorption losses. Here, based on the topological protection of electromagnetic waves, we demonstrate nonlinear generation and topologically tuned confinement of THz waves in an engineered lithium niobate chip forming a wedge-shaped Su–Schrieffer–Heeger lattice. Experimentally measured band structures provide direct visualization of the THz localization in the momentum space, while robustness of the confined mode against chiral perturbations is also analyzed and compared for both topologically trivial and nontrivial regimes. Such topological control of THz waves may bring about new possibilities in the realization of THz integrated circuits, promising for advanced photonic applications.

## Introduction

The surge of interest and the development of reliable terahertz (THz) technology is driven by the high demand for applications in, e.g., wireless communications^1,2^, signal processing^3–5^, biosensing^6,7^ and non-destructive evaluation^8^. However, the lack of integrated functional devices in the THz range, including THz emitters and detectors, has limited their applications. In addition, it has always been a challenge to guide and manipulate THz waves due to unavoidable losses arising from the critical features of the THz spectrum. Therefore, there have been tremendous efforts in exploring different designs and approaches for THz sources and integrated THz devices using a variety of platforms, including metamaterials^3,5^ and nonlinear metasurfaces^9^, surface plasmonic waves^10^, nonlinear wave mixing in ionic crystals^11^, as well as time-domain integration of THz pulses^12^.

Topological photonic systems, featuring both edge and interface modes immune to disorder and impurities^13–18^, have shown excellent potential for many applications such as in topological insulator lasers^19–21^. However, limited by material platforms and characterization methods, most research on topological photonics thus far has been focused on either the microwave or optical wave regimes. Recently, the concept of topological phase of light has been explored for implementation in THz waveguides and circuits, and for the development of future THz communications^22–24^. In particular, it has been shown that, by building a domain wall between two structures with opposite valley-Chern numbers, THz waves can be transmitted through sharp bends without significant losses. This remarkable property is due to the topological protection of the valley-Hall edge states^23,25^. These topological photonic structures are expected to be highly beneficial towards the realization of compact and robust THz functional devices.

**Fig. 1:**
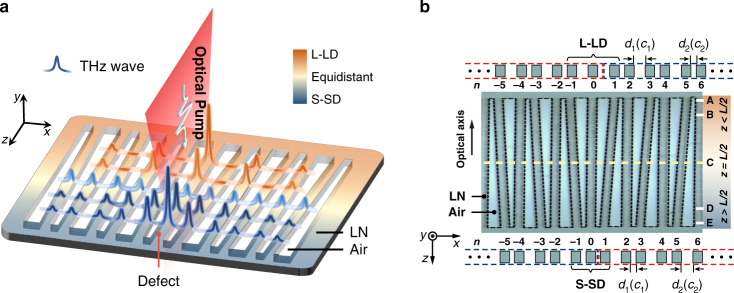
Experimental realization of topologically controlled THz localization. **a** Illustration of nonlinear generation and confinement of THz-waves in an SSH-type microstructure. The LN structure undergoes a transition from L-LD, through equidistant, to S-SD regions along the +*z-*axis, illustrated by colors shaded from orange into blue. The polarization of the THz electric field and that of the optical pump beam are all along the direction of the LN crystalline axis (*z*-axis). **b** Microscope image of the LN array structure fabricated by fs-laser writing. The thickness of the LN chip is 50 μm in the *y*-direction. The total length of the microstructure along the *z*-direction is $L = 6\,{{{\mathrm{mm}}}}$. *d*_1_ and *d*_2_ are the spacings between neighboring LN stripes corresponding to the coupling coefficients *c*_1_ and *c*_2_, respectively. At the dashed yellow line, *z* = *L*/2 and *d*_1_ = *d*_2_ = 55 μm, which leads to an equidistant structure. The SSH lattice contains an L-LD ($d_1 \,>\, d_2$) region above the line and an S-SD ($d_1 \,<\, d_2$) region below the line. A–E denote the locations corresponding to $z = 0$, *L*/8, *L*/2, 7*L*/8, *L*. Here *n* numbers the LN waveguides (lattice sites). Red and blue dashed lines mark the topologically nontrivial and trivial parts of the SSH lattice

In this work, we propose and demonstrate a scheme for nonlinear generation and topologically tuned confinement of THz waves, fully realized in a single lithium niobate (LN) photonic chip. Such a scheme relies on a judiciously designed photonic microstructure—a one-dimensional Su–Schrieffer–Heeger (SSH) lattice^26,27^ consisting of LN waveguide stripes with wedge-shaped air gaps, amenable to undergo topologically trivial to nontrivial transitions. The structure is fabricated by means of a femtosecond-laser writing technique, with a topological defect at the central interface (Fig. 1a, b). Using a pump-probe experiment, we directly map the THz-field, showing tunable confinement along the chip with respect to the variation of the photonic structure geometry. We obtain the band structures by mapping the signals measured via time-resolved spectroscopy into the momentum space, where nontrivial topological states are clearly identified. The robustness of edge states against chiral perturbations is theoretically analyzed, showing excellent agreement with experiments. Our results provide clear evidence of THz-wave confinement due to topological protection.

## Results

A standard technique for THz-wave generation is based on the optical rectification (OR)^28,29^ that may be induced by femtosecond laser pulses in second-order nonlinear crystals^30–32^. Assuming mainly electronic contribution, the nonlinear polarization is described by^29^

1 $$P(\Omega ) = \mathop {\int}\limits_{\omega - {{\Delta }}\omega /2}^{\omega + {{\Delta }}\omega /2} {\chi ^{\left( 2 \right)}\left( {\Omega ;\omega {^\prime} + \Omega , - \omega {^\prime} } \right)E_{\rm {p}}\left( {\omega {^\prime} + {{\Omega }}} \right)E_{\rm {p}}^ \ast \left( {\omega {^\prime} } \right){{{\mathrm{d}}}}\omega {^\prime} }$$

where $\chi ^{\left( 2 \right)}$ represents the second-order nonlinear susceptibility. *ω* and Δ*ω* are the central frequency and the spectral width of the pump laser pulse, respectively, and Ω represents the frequency of the generated THz waves. $E_{\rm {p}}\left( {\omega {^\prime} + \Omega } \right)$ and $E_{\rm {p}}^ \ast \left( {\omega {^\prime} } \right)$ are the electric fields of the pump laser corresponding to different frequency components. The electric field *E*(Ω) of the generated THz waves is proportional to the nonlinear polarization *P*(Ω). Over the past decades, numerous techniques have been developed to enhance THz generation efficiency, achieve narrow THz bandwidth, and decrease THz-wave decay in LN crystals^31–33^. In particular, it has been recently shown that giant enhancement of the optical nonlinearity can be achieved at THz-frequencies by stimulated phonon-polaritons in LN crystals pumped by a femtosecond laser pulse, where ionic contributions to light–matter interaction become significant^11^. While the underlying mechanisms under different excitation conditions merit further investigation, it is experimentally evident that tunable THz pulses can be generated in nonlinear LN crystals by means of ultrashort laser pulses in the range from a few tenths of THz to a few THz^11,31,32^.

Despite the rapid development in this field, new techniques are still desirable for THz-wave localization and confinement, as the THz waves typically spread out and decay quickly due to both loss and diffraction in standard device geometries. Here we employ an SSH-type photonic lattice etched in an LN chip to achieve tunable topological THz-wave localization, with a frequency range of 0.1–0.8 THz in accordance with the specific experimental parameters discussed later and detailed in the Supplementary Information. The one-dimensional SSH lattice, serving as a prototypical topological model, has been widely demonstrated for instance in photonics^27,34^ and plasmonics^35^. Moreover, such a model has been successfully tested for robust generation of entangled photon pairs^36^, enhancement of nonlinear harmonic generation^37,38^, realization of topological lasing^39^ and non-Hermitian topological states^40,41^, yet not in the THz wavelength regime. The SSH-type structure established in our experiment possesses a central interface topological defect^34,40,41^, formed by fs-laser-writing of LN waveguide arrays with varying spacing along the chip (Fig. 1b). The distances between neighboring LN stripes are given by

2 $$d_1 = d_{10} - z\delta d/L,\quad d_2 = d_{20} + z\delta d/L$$

where $L = 6\,{{{\mathrm{mm}}}}$ is the total length of the LN chip along the *z*-axis. The distance *z* is measured from the top of the chip (Fig. 1b), and the dimer structure at the top features *d*_10_ = 80 μm and *d*_20_ = 30 μm. Here *d*_1_ and *d*_2_ indicate the distances between adjacent stripes, which in turn determine the coupling coefficients *c*_1_ and *c*_2_, respectively. In particular, a larger distance *d* leads to a weaker coupling *c*. Moreover, when *δd* is set to nonzero (e.g., 50 μm in our case), we can have a “tunable” SSH-type lattice because the dimerization changes along the *z*-axis. As seen in Fig. 1b, the defect is located at the center (*n* = 0), but it varies from a *long-long defect* (L-LD) (when *z* < *L*/2) to a trivial equidistance without defect (at *z* = *L*/2), and then to a *short-short defect* (S-SD) (when *z* > *L*/2), thereby we can achieve different topological phases for the SSH structure^34^ in these three different regions (illustrated by different colors in Fig. 1a).

**Fig. 2:**
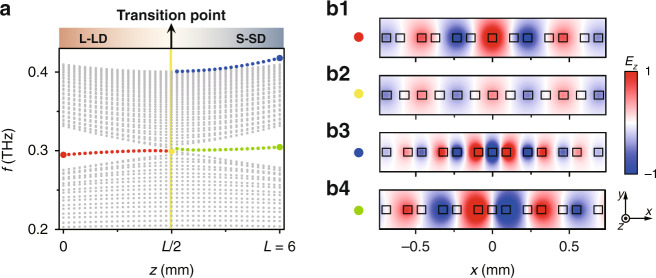
Eigenvalues and representative eigenmode distributions in the SSH-type LN topological structure. **a** Calculated eigenvalue distribution of the microstructure along the *z*-axis. The yellow line represents the equidistant structure at *z* = *L*/2 (*d*_1_ = *d*_2_ = 55 μm), which marks the phase transition point. The left side of the yellow line (*z* < *L*/2) is the L-LD region, where topological defect modes are denoted by red dots. The right side (*z* > *L*/2) indicates the S-SD region, where topologically nontrivial and trivial defect modes are marked by green and blue dots, respectively. Gray dots represent the bulk modes. **b1** Topological defect mode around 0.3 THz in the L-LD structure at *z* = 0. **b2** The mode around 0.3 THz in the equidistant structure at *z* = *L*/2. **b3**, **b4** Topological trivial mode around 0.42 THz (**b3**) and nontrivial mode around 0.3 THz (**b4**) in the S-SD structure at *z* = *L*

To better appreciate the difference in the topological characteristics, we calculate the eigenvalues of the SSH lattice in different regions along the *z*-axis and plot them in Fig. 2a. The yellow line in the middle denotes equidistance, which corresponds to the topological phase transition point. Before this line, we have *z* < *L*/2 and $d_1 \,>\, d_2,$ hence the lattice belongs to a topological structure with an L-LD at the interface (Fig. 1b). The topological property of the SSH model can be evaluated by their corresponding Zak phase^42^, where the left part of L-LD illustrated on top of Fig. 1b leads to a nonzero Zak phase while the right part contributes to a trivial Zak phase. As a result, nontrivial topological defect modes with eigenvalues residing in the middle of the gap are found at a frequency of ~0.3 THz using the parameters above (see red dots in Fig. 2a). The defect mode shown in Fig. 2b1 is located at the interface of two sub-lattices of the L-LD structure, where the mode profile distributes only at the even-numbered lattice sites labeled in Fig. 1b, featuring alternating opposite phase. Such an amplitude/phase distribution of the defect mode indicates the signature of nontrivial topology protected by the chiral symmetry of the SSH lattice^18,26,27^. Right at the transition point *z* = *L*/2, *d*_1_ = *d*_2_ = 55 μm, the L-LD structure is transformed, according to Eq. (2﻿), into a simple 1D equidistant lattice (see the yellow dashed line in Fig. 1b) with a closed gap in the band structure (see the yellow line in Fig. 2a). The structure corresponding to this point has no more dimerization and thus turns into a trivial periodic lattice. As such, at 0.3 THz the mode distributes across the whole lattice (no localized edge state) (Fig. 2b2). This point marks the occurrence of a topological phase transition.

When *z* > *L*/2, this lattice structure turns into an S-SD regime and it becomes topologically nontrivial again, similar to the L-LD regime except that now there are three LN stripes (lattice sites) closely spaced at the interface forming the “short-short defect” (see the bottom of Fig. 1b). Therefore, a topological defect mode also arises in the S-SD structure at around 0.3 THz (see green dots in Fig. 2a). However, the mode in this case is solely distributed at odd-numbered lattice sites with alternating opposite phases, but no field distribution is present on the central defect site (*n* = 0). Moreover, apart from the topological defect mode supported in the S-SD structure, there exists a trivial mode located around 0.42 THz. Such a trivial mode peaks on the defect site and occupies all lattice sites (see blue dots in Fig. 2a and b3). Most importantly, as we shall show later, it has no topological protection, in contrast to the nontrivial defect mode at around 0.3 THz. Due to such difference in the mode distribution, the excitation condition in the experiment determines if a trivial or a nontrivial mode would be excited in the S-SD end of the chip. Thus, along our specially patterned LN lattice structure, the confinement of the THz waves generated by the nonlinear excitation can be fine-tuned by scanning the pump beam through the structure to undergo a topological phase transition.

**Fig. 3:**
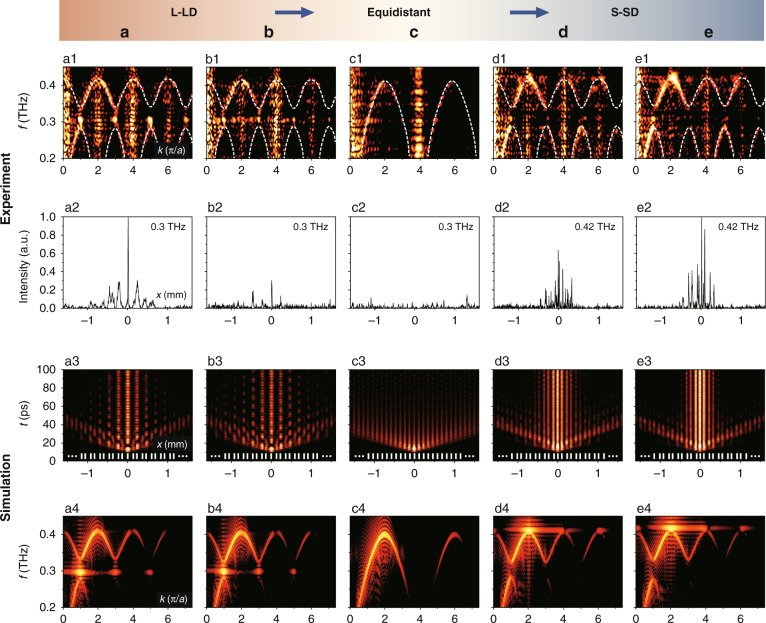
Experimental (top two rows) and numerical (bottom two rows) demonstrations of topologically controlled THz confinement in the LN chip from L-LD, through equidistant, to S-SD regions of the wedge-shaped SSH photonic lattice. **a**–**e** correspond to locations (A–E) marked in Fig. 1b. **a1**–**e1** Measured spectra at the corresponding positions. **a2**–**e2** Energy distribution of the modes showing different confinement of the generated THz waves in the LN chip. **a3**–**e3** Simulated *x*−*t* diagrams showing the THz waves evolution in different regions, where **a4**–**e4** are the corresponding spectra. The lattice sites are illustrated by white tick marks in **a3**–**e3**, and *a* in (**a1**, **a4**) is the lattice constant for the corresponding L-LD structure

In order to realize the proposed THz field manipulation, we perform a series of experiments with a typical pump-probe setup^11^ to obtain both the dispersion curves of the structure at different *z*-positions (Fig. 3, first row) and the energy distributions of the confined modes (Fig. 3, second row). In our tests, a femtosecond pump beam (800 nm central wavelength, 120 fs pulse duration, 1 kHz repetition rate) is focused onto the center defect of the LN chip via a cylindrical lens (see Fig. 1a), so as to generate THz waves via the nonlinear OR process. The evolution and confinement of the THz waves take place in the chip rather than in free space. Therefore, our scheme can be extended to integrated topological circuits and implemented in compact THz devices. For THz wave detection, the lateral propagation of waves (along the *x*-direction in the chip plane) can be directly observed using time-resolved imaging by monitoring the refractive index change induced by the THz waves and using a phase contrast imaging technique^11^. We mention that there are several other ways to observe the band spectra of structures such as angle-resolved transmission measurements^43^, energy-resolved photoluminescence^44^ and polarization-resolved momentum-space imaging spectroscopy^45^. In this work, the time delay between pump and probe pulses is tuned before launching them onto the LN chip. Then, we measure spatiotemporal evolutions of the THz field and thus acquire the related dispersion curves by performing a two-dimensional (2D) Fourier transform on the *x*-*t* diagrams (see more details in the Supplementary Information).

Experimental results obtained with the LN sample shown in Fig. 1b are summarized in the top two rows of Fig. 3. In the L-LD region ($z \,<\, 3\,{{{\mathrm{mm}}}}$), the topological defect mode appears around 0.3 THz, clearly identified in the middle of the bandgap (Fig. 3a1). Close to the top (*z* = 0, location A in Fig. 1b), most energy at this THz frequency is confined in the center defect with only weak sidelobes populating the even-numbered sites (Fig. 3a2). This strong localization corresponds to a topological defect mode in the L-LD structure, in good agreement with the calculation shown in Fig. 2b1. Closer to the center, the localization tends to vanish since the SSH structure turns into the weakly nontrivial region (Fig. 3b). At the middle (equidistance) position ($z = 3\,{{{\mathrm{mm}}}}$, location C in Fig. 1b), the structure undergoes a critical topological phase transition into a trivial lattice, so that the THz field spreads into the bulk and the intensity localization disappears (Fig. 3c2). In this case, since the lattice period is halved (no dimerization), the range of the Brillouin zone is doubled, and no defect mode can be identified in the spectrum (Fig. 3c1). Moving further into the S-SD region ($z \,>\, 3\,{{{\mathrm{mm}}}}$), we observe again strong localization but at a frequency around 0.42 THz. Close to the bottom ($z = 6\,{{{\mathrm{mm}}}}$, location E in Fig. 1b), the localized THz waves at this frequency have intensity peaks in all lattice sites next to the center defect (Fig. 3e2), indicating that the trivial defect mode (as in Fig. 2b3) in the S-SD structure is excited. Even though the input Gaussian-like pump does not favor the excitation of a nontrivial defect mode in this case, featuring a dipole-like structure near the center defect (as in Fig. 2b4), the phase difference associated to THz waves spreading away from the center stripe (caused by a slight tilt of the pump beam) leads to a minor trace of the topological state around 0.3 THz in the experiments. The phase difference caused by the tilting of the pump beam can be further used to tune the population of the topological mode, meriting further investigations (see the Supplementary Information for more details).

The above experimental results clearly demonstrate that the generated THz waves can be strongly confined near the center defect in both the L-LD and S-SD regions (Fig. 3, second row) away from the transition point (marked in Fig. 2a). The significant difference between these two regions is that the localization of 0.3 THz in the L-LD (marked as a, b in Fig. 3) has topological protection, whereas the observed localization of 0.42 THz in the S-SD (marked as d, e in Fig. 3) results from trivial defect modes. These observations are further corroborated by numerical simulations presented in the bottom two rows of Fig. 3, where Fig. 3a4–e4 are obtained by performing 2D Fourier transform on the *x*-*t* diagrams in Fig. 3a3–e3. There seems to be a difference between experimental and simulated results if one compares the top and the bottom rows. This is simply due to the instability of the laser which causes background noise, leading to spurious vertical lines in the spectra that also indicate the positions of the Brillouin zone edges (see more details in the Supplementary Information). Apart from that, simulations show good agreement with the experimental results. Upon nonlinear excitation, THz waves are generated and switched from a topological defect mode to a bulk mode, and then to a trivial defect mode, exhibiting tunable confinement and varying THz topological mode population along the LN chip (see the Supplementary Information for more details).

**Fig. 4:**
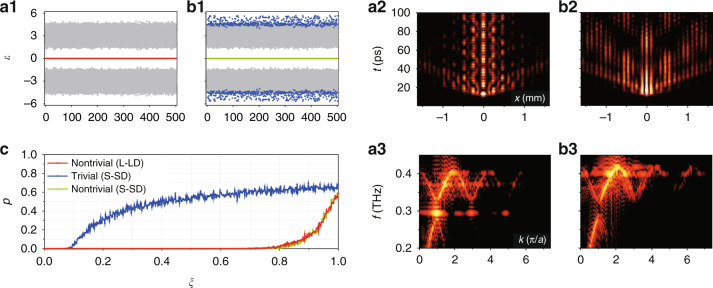
Distinction between topologically nontrivial and trivial defect modes under chiral perturbations. **a1** Calculation of the eigenvalue distribution *ε* under 500 sets of off-diagonal perturbations in the L-LD structure. The red dots (forming a line) represent the eigenvalues associated to the topological mode and the gray dots show the distribution of the bulk modes. **a2** Simulation of the *x−t* diagram for the central defect excitation under perturbations. **a3** The corresponding spectrum of (**a2**). **b1**–**b3** have the same layout as (**a1**–**a3**) but for the S-SD structure, where green and blue dots denote nontrivial and trivial defect modes, respectively. **c** Plot of *p* versus perturbation strength *ξ*, where $p = n_{{{{\mathrm{bulk}}}}}/n_{{{{\mathrm{all}}}}}$, with $n_{{{{\mathrm{bulk}}}}}$ defined as the number of perturbation sets that result in coupling of the trivial defect mode with the bulk modes and $n_{{{{\mathrm{all}}}}}$ as the total number of perturbation sets (in this case $n_{{{{\mathrm{all}}}}} = 500$). Red and green lines illustrate the nontrivial modes in the L-LD and S-SD structures, respectively, while the blue line is for the trivial defect mode in the S-SD structure

Let us further analyze the distinction between topologically nontrivial and trivial defect modes, using the following tight-binding SSH model with an interface defect:

3 $$\begin{array}{l}H = {c_{1}}\left({\mathop{\sum}\limits_{n \in N_{+}} {(1 + \xi_{n + 1})a_{n}^{\dagger} a_{n + 1}} + \mathop{\sum}\limits_{n \in N_{-}} {(1 + \xi _{n - 1})a_{n}^{\dagger} a_{n - 1}} } \right)\\\qquad + \,c_{2}\left({\mathop{\sum}\limits_{n \in N_ {+}} {(1 + \xi_{n + 2})a_{n + 1}^{\dagger} a_{n + 2}} + \mathop{\sum}\limits_{n \in N_{-}} {(1 + \xi_{n - 2})a_{n - 1}^{\dagger} a_{n - 2}}} \right)\\\qquad +\,{\rm {\it h.c.}}\\\qquad N_{+} = 2N,N_{-} = -2N,N = (0,1,2,3 \ldots)\end{array}$$

where *a*_*n*_ ($a_n^{\dagger}$) is the annihilation (creation) operator in the *n*^th^ site of the lattice labeled in Fig. 1b. $\xi _n$ is the perturbation ratio (defined as the added perturbation over unperturbed coupling coefficients). *c*_1_ and *c*_2_ describe the coupling coefficients between the LN stripe waveguides spaced by *d*_1_ and *d*_2_ in Fig. 1b, respectively. The coupling strength can be effectively tuned by changing the distance between neighboring stripes, where a smaller spacing leads to a larger coupling. When $\xi _n = 0$, $d_1 \,>\, d_2$ results in $c_1 \,<\, c_2$ in the L-LD case, however $d_1 \,<\, d_2$ results in $c_1 \,>\, c_2$ in the S-SD case. Since the salient characteristics of a topological mode are represented by its intrinsic robustness against perturbations, we add chiral perturbations ($\xi _{ - n} = \xi _n$) on all off-diagonal terms of the Hamiltonian in Eq. (3) (i.e., on all coupling coefficients without breaking the chiral symmetry^41^). To perform a quantitative analysis, we set $c_1 = 1$, $c_2 = 3$ for the L-LD, and $c_1 = 3$, $c_2 = 1$ for the S-SD, and add 500 sets of perturbations with $\max \left( {\xi _n} \right) = 30\%$. For the L-LD case, the eigenvalue of a topological defect mode is robust and isolated from the bulk modes under perturbations (Fig. 4a1). In the S-SD structure, even though a topological mode is still robust, the trivial defect mode excited in Fig. 3d, e is severely affected by perturbations (Fig. 4b1). Direct comparison of numerically simulated *x*−*t* diagrams and the differences in the corresponding spectra of the THz field between the L-LD and the S-SD structures under perturbations also confirm the features typically associated to topological protection, as shown in Fig. 4a2, a3 and b2, b3. Therefore, the topological structures can be employed to suppress or eliminate scattering loss and decay of the THz waves. As shown in Fig. 4c, even at a high perturbation ratio ($\max \left( {\xi _n} \right) = 60\%$), the THz field confinement still persists due to the robustness of the nontrivial topological mode. On the other hand, under the same experimental conditions (see the Supplementary Information for detail), half of the perturbation sets show coupling between the trivial defect mode and the bulk mode.

## Discussion

While THz-wave nonlinear generation and topological confinement can be completely independent processes, the underlying physics arising from the interplay between optical nonlinearity and topological protection certainly calls for further investigation. As discussed in the Supplementary Information, the initial nonlinear excitation can affect both topological mode population and tuning of the generated THz waves in a given photonic structure. In addition, the frequency range of the THz waves can also be adjusted by modifying the spot size of the excitation beam, and the frequency position of the localized states can be tuned by scaling the dimensions of the topological structure etched on the chip. It should be pointed out that the low coupling efficiency between THz waves and structures has been a long-standing problem. By enabling controlled nonlinear THz-wave generation and localization in a single chip, our LN topological platform may bring about a new approach to overcome this problem.

In conclusion, we have demonstrated a scheme for nonlinear generation and topologically tuned THz-wave confinement in a single photonic chip. On-chip topological phase transitions occur along the *z*-direction, in turn leading to localization/delocalization of the nonlinearly generated THz waves. Our theoretical analysis found good agreement with experimental observations and further substantiated the distinctive features of the THz topological states under chiral perturbations. This work offers a flexible and convenient way to tune the confinement as well as the topological properties of THz waves on demand, which may open an avenue towards the implementation of versatile, stable and compact THz photonic integrated circuits for various applications^2,8,23,46,47^. In the future, the study of different types of topological phenomena, such as Weyl points and topological phases in higher orders and synthetic dimensions, as well as topological phases in non-Hermitian systems, may be extended to the THz frequency range. In particular, intertwining of topological photonic structures and nonlinear effects has led already to nonlinear tuned topological states^38,48–52^, topological insulator lasers^19–21^, and topologically enhanced frequency conversion^37,53,54^. Much could be expected when the interplay of nonlinearity and topology is extended to the THz wave regime. These studies may bring about intriguing possibilities on how to judiciously manipulate the generation and propagation of THz waves, which may eventually contribute to the development of future topology-driven photonic technologies^18–24,55–57^.

## Materials and methods

Details about materials used for sample fabrication, experimental setup and techniques, and methods used for numerical simulations can be found in the Supplementary Information.

## Supplementary information

Suppl Mater
