# Supplementary material for: Topologically tuned terahertz confinement in a nonlinear photonic chip
Source: Light Sci Appl. 2022 May 23;11:152. doi: 10.1038/s41377-022-00823-7 (PMC9126941; doi:10.1038/s41377-022-00823-7)
Supplement: Supplementary file 1 — Suppl Mater [file 41377_2022_823_MOESM1_ESM.docx]

**Supplementary Information for**

**Topologically** **tuned terahertz confinement in a nonlinear photonic chip**

Jiayi Wang^1,*^, Shiqi Xia^1,*^, Ride Wang^2,*^, Ruobin Ma^1^, Yao Lu^1^, Xinzheng Zhang^1,3^‡, Daohong Song^1,3^, Qiang Wu^1,3^, Roberto Morandotti^4^, Jingjun Xu^1^‡, Zhigang Chen^1,3^‡

*^1^The MOE Key Laboratory of Weak-Light Nonlinear Photonics, TEDA Institute of Applied Physics and School of Physics,*

*Nankai University, Tianjin 300457, China*

*^2^Innovation Laboratory of Terahertz Biophysics, National Innovation Institute of Defense Technology, Beijing 100071, China*

*^3^Collaborative Innovation Center of Extreme Optics, Shanxi University, Taiyuan, Shanxi 030006, China*

*^4^INRS-EMT, 1650 Blvd. Lionel-Boulet, Varennes, Quebec J3X 1S2, Canada*

^*^*These authors contributed equally to this work.*

[‡*zxz@nankai.edu.cn*](mailto:‡%20zxz@nankai.edu.cn)*,* [*jjxu@nankai.edu.cn*](mailto:jjxu@nankai.edu.cn)*, zgchen@nankai.edu.cn*

**Notes 1: Sample fabrication and experimental setup**

The lithium niobate (LN) microstructure used in our experiments is fabricated by means of a femtosecond-laser direct writing technique^1^. The schematic diagram of the experimental setup for sample fabrication is shown in Fig. S1a. An fs-laser pulse (800 nm central wavelength, 500 μJ per pulse, 1 kHz repetition rate, 120 fs pulse duration) passes first through a power adjustment system composed of a half-wave plate (HWP) and a Glan-Taylor prism, and is then focused on a polished *x*-cut LN slab through a beam splitter and an objective lens. The scanning path of the etching process sketched in Fig. S1b is used to obtain a structure hollowed-out along the *y*-axis. In order to monitor both processing and its quality in real time, a cold light source is placed at the other end of optical path to irradiate the sample and then the sample is imaged onto a CCD camera. During the etching process, we adopt a cyclic scanning method in which the focal point of the laser is moved from the front to the back surface of the LN chip in order to obtain a better controlled etching for the sample. Moreover, in order to ensure the cleanness of the surfaces and boundaries of the structure, 2 μm-thick SiO_2_ layers are coated on both LN surfaces by way of magnetron sputtering. After processing, the LN samples are cleaned with hydrofluoric acid to get rid of SiO_2_ residuals and meanwhile remove most of the debris from processing. A microscope image of the fabricated structure (LN stripes with wedge-shaped air gaps), corresponding to the Su-Schrieffer-Heeger (SSH) photonic lattice used in our experiments, is shown in Fig. S2. Such structure has a “defect” waveguide in the center and varies from a long-long defect (L-LD, top half) through an equidistant (middle) and then to a short-short defect (S-SD) region (bottom half).


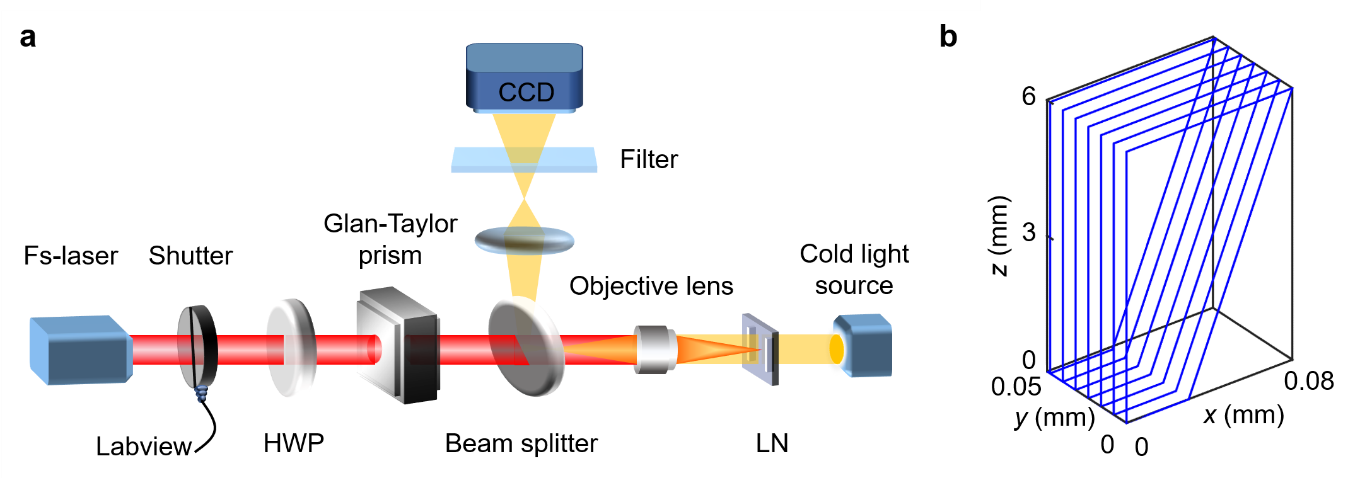


Fig. S1. Schematic diagram of the fs-laser writing technique for sample fabrication. (a) Sketch of the experimental setup. (b) Illustration of the cyclic scanning path.


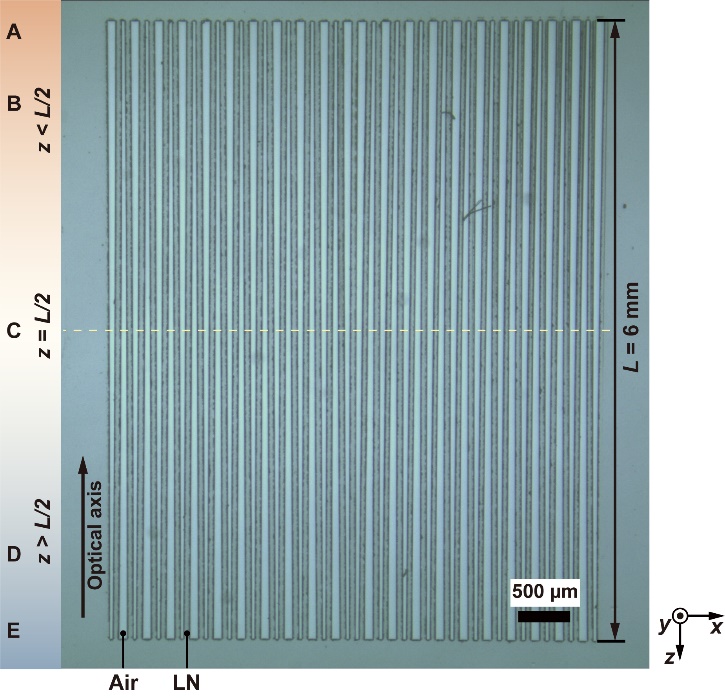


Fig. S2. Microscope image of the SSH photonic structure (array of LN stripes separated by wedge-shaped air gaps) fabricated by means of an fs-laser writing technique. The optical axis of the LN is placed along the $z$ direction as shown by the arrow. The thickness of the LN chip is 50 μm along the *y*-axis. The total length of the microstructure along the $z$-direction is $L=6 \mathrm{mm}$. At the dashed yellow line, $z=L/2$ leads to an equidistant structure. Such structure features an L-LD ($z<L/2$) region above the line and an S-SD ($z>L/2$) region below the line. A, B, C, D and E denote the locations along $z$-direction where the experimental data were taken as described in the main text.

A schematic of the pump-probe experimental setup is shown in Fig. S3. The fs-laser pulse from a Ti: sapphire regenerative amplifier (as specified above) is divided into a pump beam (carrying 90% of the total energy) and a probe beam (carrying 10% of the total energy) by a beam splitter. The pump beam is sent through a mechanical delay line, and then line-focused on the defect site by a cylindrical lens. This allows to generate a linearly polarized THz field via optical rectification (OR) in the LN chip, as illustrated in the inset^2^. For what concerns the LN nonlinear crystal, the second-order nonlinear coefficient can be expressed as a third-rank tensor relating the amplitude of the nonlinear polarization to the product of the THz field amplitudes. Since the *d*_33_-tensor element (along the *z* axis) is the largest nonlinear coefficient in LN, it is clearly desirable to couple the polarization of the incident optical pump pulse along the *z* direction in order to produce the largest THz *E*-field amplitude. Therefore, to get an optimum THz signal, we used a *z*-polarized pump beam and focused it, via a cylindrical lens, into the LN slab, so as to produce linearly polarized THz waves in experiment.

The probe beam is frequency-doubled to 400 nm by a BBO crystal, spatially filtered and then expanded to illuminate the entire structure at normal incidence. Due to the electro-optical effect, the propagation of THz waves in the LN microstructure along the *x* axis can lead to variations in the refractive index of the sample, and then give rise to a change in the phase of the probe beam. The quantitative relationship is as follows:

$$\Delta\varphi\left( x,z,t \right)=2\pi\frac{l}{\lambda}\Delta n\left( x,z,t \right)=2\pi\frac{l}{\lambda}\frac{n_{e}^{3}\gamma_{33}}{2}E_{\mathrm{THz}}\left( x,z,t \right) (S1)$$

where $l$ is the thickness of the LN chip, $\lambda$ is the wavelength of the probe beam, $n_{e}$ is the extraordinary refractive index for the probe in the LN sample, $\gamma_{33}$ is the electro-optical coefficient, and $E_{\mathrm{THz}}$ is the average THz electric field. In this way, the generated THz waves propagating along the LN chip can be quantitatively measured.

As the CCD camera can only capture the intensity information, the phase change of the probe beam needs to be converted by phase contrast imaging into intensity information^3^. A phase plate is put on the Fourier plane of the first lens, which can induce a $\pi/2$ phase modulation for the $\text{400 nm}$ probe wavelength. The phase plate is created by using a fused silica on a substrate ($25\times25 mm^{2}$-surface area, $1 \mathrm{mm}$-thickness). A 222 nm layer of SiO_2_ with a refractive index of about 1.45 at $\text{400 nm}$ is first spin-coated on the substrate. Then, a $35\times35 \mu m^{2}$ area at its center is removed by electron beam lithography. Accordingly, the phase plate possesses a flat surface with a central square area depressed by 222 nm, leading to a $\pi/2$ phase modulation.

By varying the time delay between the pump and probe pulses to the sample, we can obtain the whole spatiotemporal evolution of the THz field, and then acquire the dispersion spectrum by means of a two-dimensional Fourier transform to intuitively observe the eigenmodes in the momentum space.


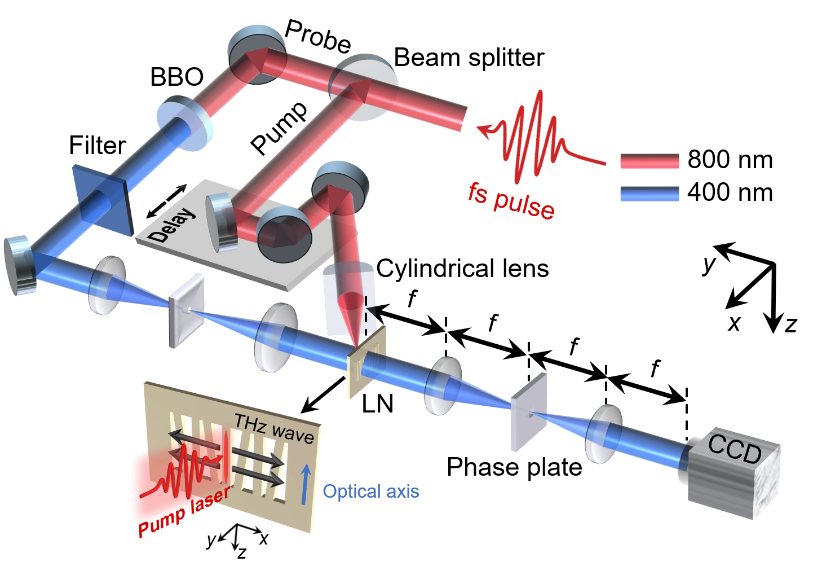


Fig. S3. Schematic diagram of the pump-probe system used in our experiment. The focal length of the cylindrical lens is $200 \mathrm{mm}$. In its phase contrast imaging section, the focal lengths of two convex lenses are both $100 \mathrm{mm}$, and the phase plate is placed at the focal plane of the first lens. The lower inset is a sketch to illustrate the generation and propagation of the THz waves along the LN chip. The optical axis of the LN is indicated by the blue arrow.

**Notes 2: Simulation with the finite-difference time-domain (FDTD) method**

In Fig. 4, we numerically simulated the spectra of the THz waves in the L-LD and S-SD LN structures under perturbations to further support theoretical calculation results obtained with the tight-binding model by using a commercial FDTD software (FDTD solutions, Lumerical). The thickness of the LN chip is set to $50 \mu m$. In the L-LD structure, the original air-gap distances between two adjacent LN stripes are set to $d_{1}=80 \mu m$ and $d_{2}=30 \mu m$, whereas in the S-SD structure, $d_{1}=30 \mu m$ and $d_{2}=80 \mu m$. Then, we add chiral perturbations on all distances between adjacent LN stripes and at the same time preserve the chiral symmetry of the system^4^. The light source, whose polarization direction is along the $z$-direction, is placed at the center defect stripe of the LN chip, with a frequency range of 0.2~0.5 THz. The boundary conditions for the simulation area along all axes are fixed as perfect matched layers. Spatiotemporal evolutions of THz field are collected by a time monitor and then the dispersion spectra are obtained by performing a two-dimensional Fourier transform of the *x*-*t* diagrams. Moreover, the maximum mesh size is set as $1 \mu m$ along all axes.

**Notes 3: Calculation of defect-bulk mode coupling under perturbation**

When we add perturbations on all couplings, all eigenvalues of the modes start to “move” in the eigenvalue spectrum. In order to evaluate the effect of perturbation on defect-bulk mode coupling, the eigenvalue differences are calculated as illustrated in Fig. S4, where the difference between the defect mode and the nearest bulk mode is $\delta\varepsilon_{1}$, and under the same perturbation, $\delta\varepsilon_{2}$ is the eigenvalue difference between two bulk modes in the vicinity of the defect mode (Fig. S4a). If $\delta\varepsilon_{2}>\delta\varepsilon_{1}$ under certain values of perturbation as denoted in Eq. 3 in main text, we consider such a defect mode as the one coupled into the bulk in this set of perturbation. The same criteria are also applied for the S-SD situation to evaluate the defect-bulk mode coupling of the trivial defect mode (Fig. S4b).


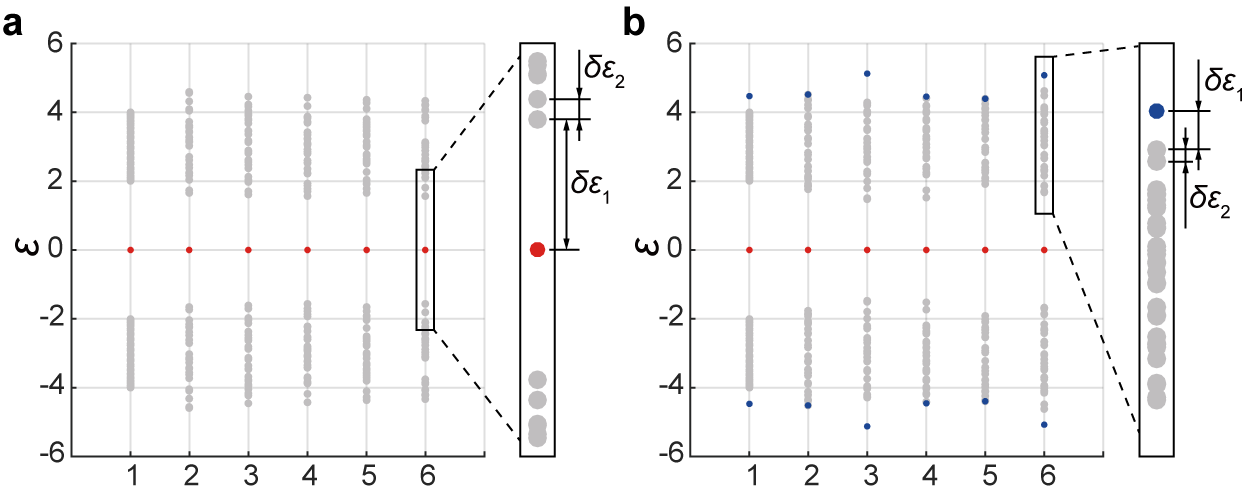


Fig. S4. Calculation of eigenvalue differences between defect and bulk modes. (a) Eigenvalue distribution of the L-LD structure under a chiral perturbation on the coupling coefficients defined in Eq. 3 of the main text, with the maximal perturbation given by $\max\left( \xi_{n} \right)=30\%$. The right column shows a zoom-in for better visualization. $\delta\varepsilon$ is the eigenvalue difference. (b) has the same layout as (a) but for the S-SD structure. First column in (a) and (b) is the corresponding eigenvalue distribution without perturbation. Nontrivial and trivial defect modes are denoted by red and blue dots, respectively, and the bulk modes are represented by gray dots.

**Notes 4:** **Idle components in experimental spectra**

Figure S5(a) depicts the dispersion diagram when there is only the probe beam but no pump beam present, which can be used as a blank control (or a reference for direct spectral comparison). The position of the vertical lines in Fig. 3a1 of the main text is exactly the same as in the blank control. The appearance of vertical lines is due to the laser instability: the instantaneous background at each moment is not exactly the same as the one we saved at the beginning of our experiments, which leads to an undefined background subtraction. Therefore, the vertical lines simply come from the two-dimensional Fourier transform of the probe beam through the SSH-type structure. The distance between adjacent vertical lines is $\Delta k=2\pi/a$, which reflects the size of the Brillouin zone.


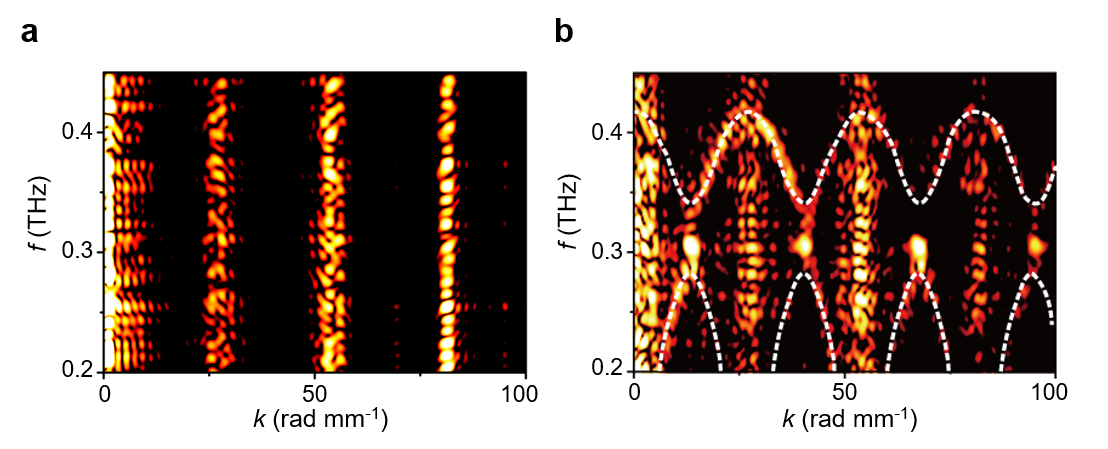


Fig. S5. (a) Dispersion spectrum in the absence of the pump beam. (b) Dispersion spectrum in the presence of the pump beam as shown in Fig. 3a1 of the main text, displayed here again for direct comparison.

When we compare the experimental (Fig. S6b) and simulated (Fig. S6a) spectra, in the S-SD structure, we can see that there is a slight trace of the topological state (the bright spot in the middle). This is because the pump beam is obliquely incident on the LN chip which leads to a different wave front for the generated THz waves at the two sides of the excitation point, further requiring different times as well as paths for the formation of stable THz waves. Therefore, the THz waves on the two sides feature different phases at the same distance away from the excitation point. Such phase difference results in a weaker excitation of the topological defect mode in the S-SD structure, since the nontrivial mode has a $\pi$ phase difference in correspondence of the stripes neighboring the defect (see Fig. 2b4 of the main text). In order to verify this, a phase difference $\pi/6$ is introduced into the left and right THz waves to simulate the situation mentioned above by the FDTD method. As can be seen in Fig. S6c, the spectrum is basically the same as the experimental one once this adjustment is made. Therefore, for further studies, the propagation direction of the pump beam can be used as a degree of freedom to tune the phase difference of the THz field at the two sides of the excitation point and lead to a tunable mode excitation, where selectively excited modes possess different topological properties.


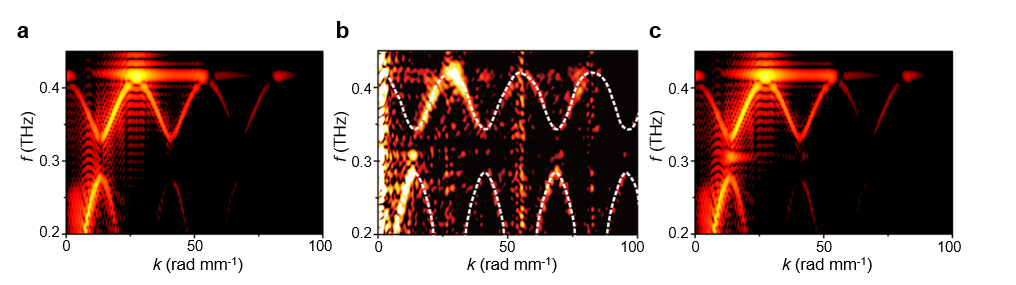


Fig. S6. (a, b) Simulated (a) and experimentally measured (b) dispersion spectra in the S-SD structure. (c) Simulated dispersion curve after considering the phase difference between left and right THz waves generated under tilted excitation, showing a trace of the topological state (bright spot in the middle of the gap) well-reproducing the experimental results.

**Notes 5: Nonlinear tuning of topological mode population at a fixed *z*-location**

In our experiments, the nonlinear generation of THz waves and their topological confinement are not completely independent. We can control the population of a topological state in the structure by altering the experimental conditions during nonlinear generation. In the process of femtosecond laser pumping to generate THz waves via OR in the LN chip, we can adjust the incident angle of the femtosecond pump laser to tune the THz confinement at a fixed *z*-location along the chip. This is because the pumping direction changes the phase difference between the generated THz waves at the two sides of the excitation point, in such a way that the population of both topologically nontrivial and trivial states is tuned. In particular, this can be used to isolate the topological mode from the trivial mode in the S-SD structure. The details are shown in Fig. S7. For the S-SD structure (Figs. S7a1-e1), the population of the topological state increases gradually as a function of the phase difference. When the phase difference is 0 (π), only the trivial (topological) state can be excited, so that topologically nontrivial and trivial states can be separated. For the L-LD structure (Figs. S7a2-e2), the population of the topological state gradually decreases as a function of the phase difference. In the latter case, when the phase difference is π, the topological state is no longer excited. These fine-tunings of topological confinement would not be possible without the presence of nonlinearity.


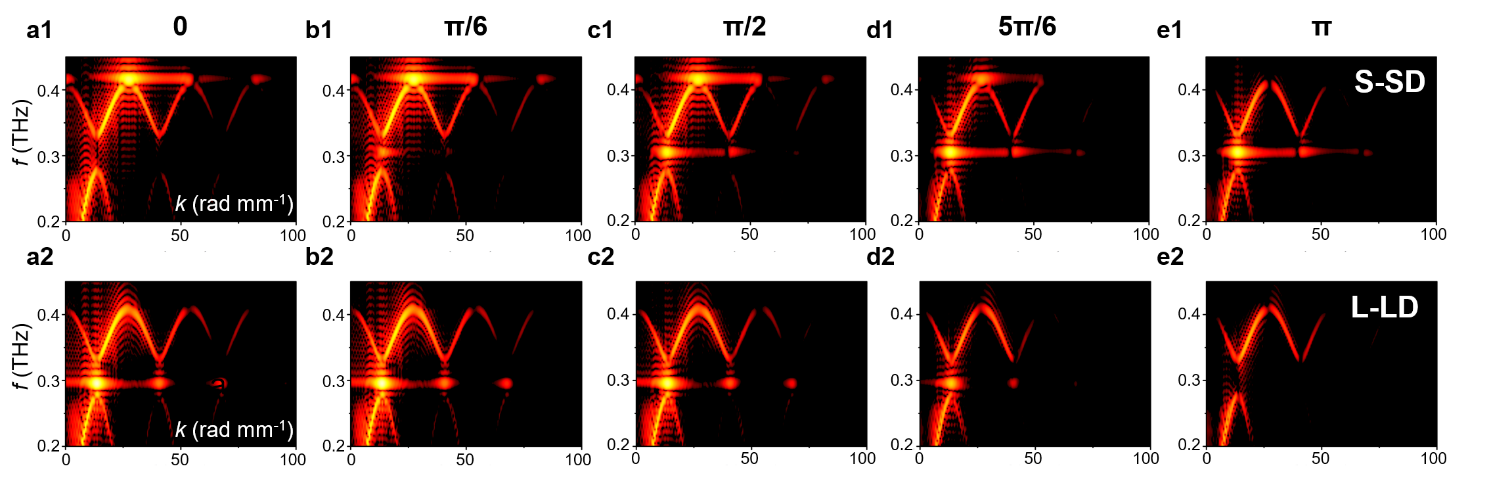


Fig. S7. Simulation of tuning between topological and trivial modes by changing the nonlinear excitation condition at top and bottom locations in the LN chip. (a1-e1)/(a2-e2) Dispersion spectra (frequency vs. wavevector) in the S-SD/L-LD structure for various phase differences between the left and right THz waves.

**Notes 6:** **Modulation of** **topological mode population along the *z*-direction**

As discussed in the main text, we demonstrate a scheme for THz nonlinear generation and topologically tuned THz-wave confinement in a single photonic chip, and illustrate the distinctive features of the THz topological states under chiral perturbations^4^. Our experimental realization benefits from the wedge-shaped SSH lattice in the LN chip. Below, we provide further numerical results to show how the confinement can be effectively tuned along the structure, in agreement with the experimental conditions presented in the main text, i.e., nearly perpendicular excitation so the phase difference illustrated in Fig. S7 is close to zero.

Under such excitation conditions, we focus on the THz wave generated as the topological mode, and examine its confinement in the center waveguide with respect to other area of the sample. Figure S8 shows the normalized population of the topological defect mode when the THz wave evolves (red line), i.e. $\langle\psi_{t}|\psi\rangle$, where $\psi_{t}$ is the topological eigenmode and $\psi$ is the generated THz waves, and the intensity ratio (blue line), i.e., $I_{center}{/I}_{all}$, which is the ratio between the intensity confined only in the central waveguide and the total intensity of the generated THz waves at different *z* positions. As seen from this figure, for the L-LD region (closer to the *z* = 0 side), away from the central topological phase transition point, the population of the topological mode tends to increase, and thus the localization of THz waves in the central waveguide becomes gradually stronger. For the S-SD region (closer to the *z* = 6 side), the localization in the center also gets stronger, but the population of the topological mode is almost close to zero, indicating that the excited mode is a trivial defect mode. Thus, our platform offers a flexible and convenient way to tune the confinement as well as the topological properties of THz waves in a single photonic chip.


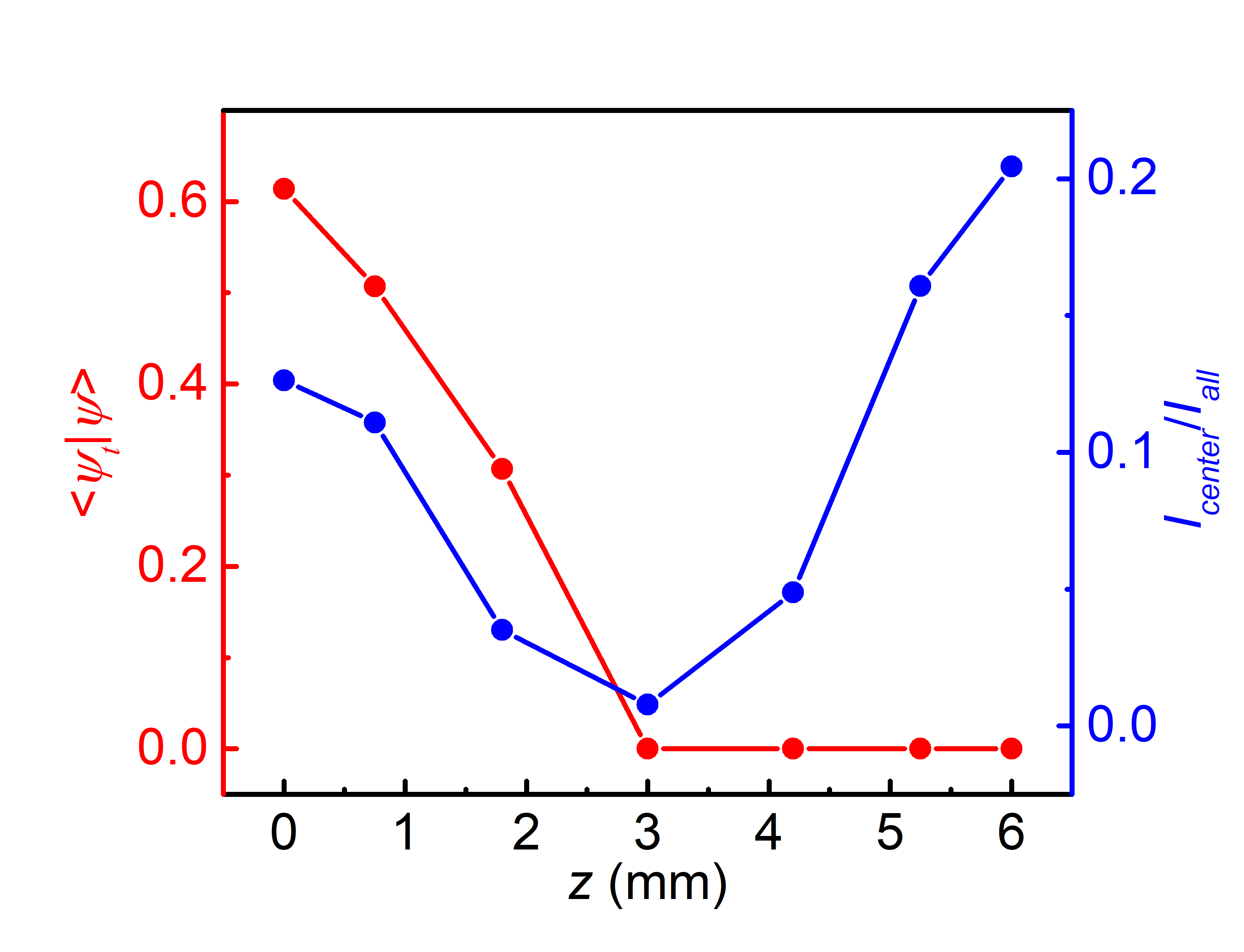


Fig. S8. Population of the excited topological mode normalized to the generated THz waves (red line) and the intensity of the THz wave in the central “defect” waveguide (normalized to the total intensity of the generated THz waves) (blue line) at different *z* positions along the SSH-engineered LN chip.

1 Sivarajah, P. *et al.* Chemically assisted femtosecond laser machining for applications in LiNbO3 and LiTaO3. *Applied Physics A* **112**, 615-622, doi:10.1007/s00339-013-7833-x (2013).

2 Lu, Y. *et al.* Giant enhancement of THz-frequency optical nonlinearity by phonon polariton in ionic crystals. *Nature communications* **12**, 3183, doi:10.1038/s41467-021-23526-w (2021).

3 Wu, Q. *et al.* Quantitative phase contrast imaging of THz electric fields in a dielectric waveguide. *Optics express* **17**, 9219-9225, doi:10.1364/OE.17.009219 (2009).

4 Xia, S. Q. *et al.* Nonlinear tuning of PT symmetry and non-Hermitian topological states. *Science* **372**, 72-76, doi:10.1126/science.abf6873 (2021).
